# Savannah Meeting of the Medical Association of Georgia

**Published:** 1907-06

**Authors:** 


### EDITORIAL NOTES AND COMMENTS.

---

The Business Office of the JOURNAL-RECORD is 1 1-2 to 5 1-2 South Broad Street.

The Editorial Office is 318-319-320 The Prudential.

Address all Business Commucications to Dr. George Brown, Manager.

Make remittances payable to THE JOURNAL-RECORD OF MEDICINE.

On matters pertaining to the Editorial and Original communications, address Dr. Bernard Wolff, Atlanta.

Reprints of original articles will be furnished at cost price. Requests for the same should always be made on THE MANUSCRIPT.

We will present, postpaid, on request, to each contributor of an original article, twenty (20) marked copies of THE JOURNAL-RECORD OF MEDICINE containing such article.

---

### SAVANNAH MEETING OF THE MEDICAL ASSOCIATION OF GEORGIA.

The Fifty-Eighth Annual Session of the Medical Association of Georgia was held at Savannah April 17th to April 19th.

After prayer by the Rev. Dr. Pickard and addresses of welcome by Mayor Tiedeman aud Dr. E. R. Corson, the scientific portion of the meeting was taken up. Owing to the increased membership, the work was divided into a medical and a surgical section.

Among other papers presented were those of Dr. Wesley Taylor, Dr. J. A. Combs and Dr. Theodore Toepel.

Dr. Taylor realizing the need for some protection and care to be given the epileptic and weak minded, recommended the establishment of a State institution for this purpose. He thought that by manual training, the unfortunate lot of such defectives could be much alleviated. Dr. Allen of Milledgeville and Dr. Kime of Atlanta, cordially seconded the recommendations contained in Dr. Taylor's paper, and secured by unanimous vote the official endorsement of the Association to the plan for the establishment of such an institution.

Dr. J. A. Combs of Locust Grove, found nothing commendable in the prevalent use of the soda fount. It led to too free commingling of the sexes, to illicit traffic in intoxicants, fostered the counter-prescribing evil, favored pernicious drug habits, and was in all particulars baneful in its influenee. He thought that greater restrictions should be thrown around soda fount dispensing.

Dr. Toepel spoke of the position of exercise in medicine and surgery. His paper may be found in the May issue of this Journal.

Dr. Oertel of Augusta, reported a case of Vincent's angina. The patient, a married woman thirty-seven years of age, had suffered from the disease for three years. The glands of the neck were much enlarged, there was a ragged ulcer on the right tonsil, which was covered with a psuedo-membrane. There were cicatrices of former ulcers upon the opposite tonsil, and the pharyngeal mucosa was congested and hypertrophied. The spirochetes of Vincent were found in smears from the tonsil. Local antiseptic treatment and general tonic medication produced a rapid amelioration of all the symptoms.

Drs. Hoke and Andrews presented the records of five cases of chronic arthritis deformans which they regarded as traceable to albuminous putrefaction in the intestines as uniform improvement in symptoms followed a diet of fermented milk.

Dr. Scott of McDonough, recommended turpentine in treatment of typhoid fever. It is given in ten drop doses, and tends to check diorrhoea and hemorrhage.

Dr. Sommerfield reported a case of symptomatic cure of pulmonary tuberculosis following home treatment made up of fresh air, attention to hygiene ventilation. disinfection and a diet of milk and raw eggs.

Dr. W. H. Adams of Savannah, presented a paper defining and describing paranoia. The disease is frequently not recognized, and intelligent study should be given it to protect the public against cranks, many of whom are affected with this form of insanity.

Dr. S. A. Visanska, Atlanta, called attention to some points in infant feeding. He advised giving raw milk during cold weather, sterilizing during hot weather and pasteurizing it during the cool seasons.

Dr. H. H. Martin. in his presidential address alluded to the work of Council of Pharmacy of the American Medical Association, and the work done in exposing nostrums and frauds. He recommended enforcement of the law against abortionists; the establishment of a State Sanitarium for tuberculosis; a minimum fee of $5.50 for licensing, and the establishment of an official journal.

Dr. H. R. Slack of LaGrange, presented a paper on the treatment of cancer with methylene blue (see JOURNAL-RECORD for May).

Dr. Saliba, Savannah, in a paper on the treatment of cough in tuberculosis, recommended inhalations, dry or moist, for cough due to catarrh of the larger air passages. Cough due to laryngeal diseases requires local anaesthetics.

Dr. Jabez Jones of Savannah, presented an apparatus for the treatment of fracture of the femur.

Dr. Craig Barrow, Savannah, reported two cases of anewrismorrhaphy, one of which was successful.

Dr. E. C. Davis, Atlanta, reported a case of multiple abscess of the pelvis, pointing out the difficulties and methods of termination and of treatment.

Dr. E. R. Corson of Savannah, presented a paper on gupra-pubic prostatectomy, in which he pointed out the merits of this operation

Drr J. W. Sigman, Savannah, in his paper on surgical anaesthesia emphasized the importance of a competent anaesthetist, deploring the practice of entrusting this procedure to the inexpert.

Dr. A. P. Hanie, Hartwell, reported a case of rupture of the gravid uterus, wtth escape of its contents info the abdominal cavity. Operation saved the life of the patient.

Under election of officers, the following officers were duly elected for the ensuing year: President, M. A. Clark, Macon; Vice Presidents, R. W. Thompson, E. E. Murphey, Augusta; L. H. Jones re-elected Secretary-Treasurer,; G. R. White and H. F. Harris were elected delegates to the A. M. A. The meeting then adjourned to hold the next session at Fitzgerald, April, 1908.

Among the social diversions of the session may be mentioned a banquet at Thunderbolt and an oyster roast at Tybee Island.

The privileges of the clubs were accorded the visiting members. The session of 1907 was officially voted to have been one of the most harmonious and enjoyable in the history of the Associaion.

---

A canvass of the public schools of Baltimore shows that there are 51 children suffering from epilepsy. The school board has decided to establish two or three special classes for epileptic children, beginning with the next school year.

---

The third annual meeting of the National Association for the Study and Prevention of Tuberculosis was held at the New Willard Hotel, Washington, D. C., May 6 to 8, 1907.

---

Daniel Osiris, a multi-millionaire of Paris, has bequeathed $5,000,00o to the Paris Pasteur Institute.

